# Developing a Simulated Online Model That Integrates GNSS, Accelerometer and Weather Data to Detect Parturition Events in Grazing Sheep: A Machine Learning Approach

**DOI:** 10.3390/ani11020303

**Published:** 2021-01-25

**Authors:** Eloise S. Fogarty, David L. Swain, Greg M. Cronin, Luis E. Moraes, Derek W. Bailey, Mark Trotter

**Affiliations:** 1 Central Queensland Innovation and Research Precinct, Institute for Future Farming Systems, CQ University, Rockhampton, QLD 4701, Australia; d.swain@cqu.edu.au (D.L.S.); m.trotter@cqu.edu.au (M.T.); 2 Faculty of Science—SOLES, The University of Sydney, Camden, NSW 2570, Australia; gregmcronin@gmail.com; 3 Department of Animal and Range Sciences, The Ohio State University, Columbus, OH 43210, USA; lmoraes2719@gmail.com; 4 Department of Animal and Range Sciences, New Mexico State University, Las Cruces, NM 88003, USA; dwbailey@nmsu.edu

**Keywords:** machine learning, parturition, on-animal sensors, sheep

## Abstract

**Simple Summary:**

Near-real-time monitoring of livestock using on-animal sensor technology has the potential to improve animal welfare and productivity through increased surveillance and improved decision-making capabilities. One potentially valuable application is for monitoring of lambing events in sheep. This research reports on the development of a machine learning classification algorithm for autonomous detection of lambing events. The algorithm uses data from Global Navigation Satellite System (GNSS) tracking collars, accelerometer ear tags and local weather data. Overall, four features of sheep behaviour were identified as having the greatest importance for lambing detection, including various measures of social distancing and frequency of posture change. Using these four features, the final algorithm was able to detect up to 91% of lambing events. This knowledge is intended to contribute to the development of commercially feasible lambing detection systems for improved surveillance of animals, ultimately improving methods of monitoring during critical welfare periods.

**Abstract:**

In the current study, a simulated online parturition detection model is developed and reported. Using a machine learning (ML)-based approach, the model incorporates data from Global Navigation Satellite System (GNSS) tracking collars, accelerometer ear tags and local weather data, with the aim of detecting parturition events in pasture-based sheep. The specific objectives were two-fold: (i) determine which sensor systems and features provide the most useful information for lambing detection; (ii) evaluate how these data might be integrated using ML classification to alert to a parturition event as it occurs. Two independent field trials were conducted during the 2017 and 2018 lambing seasons in New Zealand, with the data from each used for ML training and independent validation, respectively. Based on objective (i), four features were identified as exerting the greatest importance for lambing detection: mean distance to peers (MDP), MDP compared to the flock mean (MDP.Mean), closest peer (CP) and posture change (PC). Using these four features, the final ML was able to detect 27% and 55% of lambing events within ±3 h of birth with no prior false positives. If the model sensitivity was manipulated such that earlier false positives were permissible, this detection increased to 91% and 82% depending on the requirement for a single alert, or two consecutive alerts occurring. To identify the potential causes of model failure, the data of three animals were investigated further. Lambing detection appeared to rely on increased social isolation behaviour in addition to increased PC behaviour. The results of the study support the use of integrated sensor data for ML-based detection of parturition events in grazing sheep. This is the first known application of ML classification for the detection of lambing in pasture-based sheep. Application of this knowledge could have significant impacts on the ability to remotely monitor animals in commercial situations, with a logical extension of the information for remote monitoring of animal welfare.

## 1. Introduction

There is increased interest in the development of sensing technologies to improve animal management in the extensive grazing industries [1,2]. Much of the research to date has been conducted using individual on-animal sensors such as Global Navigation Satellite System (GNSS) tracking, motion sensors (e.g., accelerometers, inertial monitoring units, pitch and roll sensors), jaw or bite sensors and physiological sensors [1,3]. In specific studies of sheep, on-animal sensor technologies have been applied to monitor various behaviours of interest, either within a particular context (e.g., lambing [4,5,6]; predation [7]; oestrus [8]) or more generally for basic behaviour recognition [9,10,11,12,13].

In the majority of sensor-based sheep research, single sensor types are applied in isolation [1]. However, given the number of available technologies and the benefits each can provide, there is merit in exploring the use of integrated monitoring systems. This is likely to be particularly valuable when a single sensor is unable to collect all the desired information, or when the use of multiple sensors improves accuracy. For example, in work by Spink et al. [14], joint GNSS and accelerometer tracking of Canada geese found the combination of the two sensor types improved the ability to distinguish behaviours of interest, compared to GNSS alone. In Dewhirst et al. [15], integration of GNSS, accelerometers and magnetometers improved the accuracy of location and distance travelled estimates of domestic dogs. The use of integrated sensors has also been explored in cattle production systems. For example, Barker et al. [16] found integrated local positioning data and accelerometers could detect changes in dairy cow feeding behaviour associated with lameness. González et al. [17] also reported on an integrated GNSS and accelerometer behaviour monitoring system, incorporating additional live weight data from remote weighing systems, to demonstrate the value for beef cattle grazing systems. One key gap in the literature is the lack of reported use of weather data in an integrated sensor approach. Weather has obvious implications for animal behaviour [18], particularly in extensive grazing systems [19], and so its exploration as a component of an overall behavioural monitoring systems is also warranted. 

While the application of sensors in a research context is important, there is growing interest in the development of these systems for commercial application [20]. In this context, sensors will require real-time or near-real-time data processing and information transfer to ensure timely operational decisions [21]. Within this near-real-time requirement sit additional concepts of online processing and edge computing. Online processing refers to the analysis of each data point as they become available, with the aim of identifying the nonconformities as soon as possible after they occur [22]. Edge computing refers to the capacity to perform some level of processing either at or near the device, without the reliance of data transfer to the cloud [23]. Although many advances have been made in near-real-time sensor systems, there are a number of practical challenges associated with their implementation [24]. For example, data transmission is an extremely power-intensive activity and selection of data deemed most relevant to analysis may be necessary [25]. Sensor type can also impact on power requirements (e.g., GNSS receivers require significant amounts of power [26]) and computational requirements can greatly impact the power supply [24]. Given these limitations, most applications of on-animal sensors, particularly in a research context, are still conducted using “store-on-board” (SOB) devices, where the data are saved on the sensor itself and only accessible after the device has been removed [21,27]. In this case, the entire dataset is usually viewed as a whole (known as “offline” processing), with previously occurring patterns detected after they occur through an examination of historical data [22]. Although obviously not directly applicable to commercial settings, SOB devices can serve as a proxy to collect sensor data for later use in simulated online scenarios, which serve to evaluate the potential for developing commercially viable products.

One potentially valuable application of sensor technology is for monitoring of parturition (lambing) events in sheep. Lambing is a critical period for the ewe and lamb, with lasting impacts on productivity and welfare [28,29,30]. Detection of lambing has implications for two key welfare outcomes for the sheep industry. Firstly, it provides an indication of ewe welfare, particularly if applied to detect abnormal parturition-related behaviour (e.g., detection of prolapse or dystocia). Secondly, welfare of the newborn can also be inferred, given experience of dystocia or even selection of an appropriate lambing site can indicate quality of mothering and early experience of the lamb [29]. Previous sensor-based research of lambing behaviour has focused on two main technologies: firstly, GNSS [4,5,6,31]; secondly, and to a lesser extent, accelerometers [32,33]. These studies have broadly proven the ability of each sensor type to detect changes in behaviour associated with lambing. However, the application of these sensors to detect a lambing event under commercial conditions in simulated “near-real-time” is yet to be explored. This process has been examined in other livestock industries including calving beef and dairy cattle [34], farrowing pigs [35,36] and for detection of stress in police horses [37]. 

In this paper, a simulated online machine learning (ML) classification algorithm for detection of parturition events in commercial grazing ewes is developed and evaluated. In this paper, a parturition event is considered to involve both behavioural changes associated with the onset of lambing, as well as the lamb expulsion itself. SOB data were used as a substitute for near-real-time sensor data and allowed for sequential processing of each data point to simulate an online processing scenario. The algorithm uses data from GNSS tracking collars, accelerometer ear tags and local weather data and hence explores the benefits of an integrated sensor approach. The specific objectives were to: (i) determine which sensor systems and features provide the most useful information for lambing detection; (ii) evaluate how these data might be integrated using ML classification to alert to a parturition event as it occurs. Within this last objective, the concept of adjusting detection criteria post-classification is explored in the context of applying the model in situations where false positives are more or less acceptable. This knowledge is intended to contribute to the development of commercially feasible lambing detection systems for improved surveillance of animals, ultimately improving methods of monitoring during this critical period.

## 2. Materials and Methods 

### 2.1. Location and Animals 

Two independent field trials were conducted at a commercial mixed enterprise on the South Island of New Zealand (43.0° S and 173.2° E) over consecutive years. Trial One was conducted from 29 September to 13 October 2017. Trial Two was conducted from 9 September to 23 September 2018. All procedures were approved by the Massey University Animal Ethics Committee (MUAEC 17/59; MUAEC 18/67).

In Trial One, 40 mixed-age Merino or Merino-cross ewes were selected from the main commercial flock. Selection was based on ewes having an expected lambing date during the experimental period (determined via ultrasound scanning as per normal farm practice). A preliminary analysis of the data from this trial has been previously published [6]. Eight of the animals used in the development of the model reported in this paper having been previously used in [6]. However, this does not confound this study as they remain part of the independent model development cohort and not the validation data set. Furthermore, in [6], analysis was only conducted using GNSS data, not accelerometer or weather data. Finally, novel metrics are included in this paper that were not explored in [6], e.g., distance to closest peer (CP). The experimental paddock was 3.1 ha and provided ad libitum access to forage and water. 

In Trial Two, 39 mixed-age Merino or Merino-cross ewes were selected from the main commercial flock. Again, selection was based on ewes having an expected lambing date during the experimental period. Of the 39 animals selected, 12 ewes have been previously used for development of the ML behaviour algorithms [10] that are applied for prediction of animal behaviour in the current study. For this reason, these animals were excluded, and their data subsequently removed from the validation dataset. 

The experimental paddock was 4.4 ha and provided ad libitum access to forage and water. 

Throughout each trial, weather data were collected by an on-farm weather station for later incorporation into the dataset. Weather data included average air temperature, average wind speed and average solar radiation recorded hourly. Hourly rainfall was also recorded.

### 2.2. Instrumentation

In both trials, experimental ewes were fitted with devices on the morning prior to study commencement. Each animal was fitted with a GNSS logger (i-gotU GT-600, Mobile Action Technology Inc., Taiwan) attached to a neck collar and an accelerometer (Axivity AX3, Axivity Ltd., Newcastle, UK) attached to an ear tag. GNSS loggers were programmed to obtain locations at 3 min (Trial One) or 2 min (Trial Two) intervals. Accelerometers were configured at 12.5 Hz and fixed with an orientation of the X-, Y- and Z-axis along the dorso-ventral (up–down), lateral (side-to-side) and anterior–posterior (forward–backward) axes, respectively. Both GNSS and accelerometer sensors were selected for use as they provide distinctly different data. GNSS records periodic location of the animal, while accelerometer sensors provide a measure of the animals’ movement at a fine scale and high temporal frequency.

In Trial One, ewes were moved to the experimental paddock after instrument attachment and remained in this location for the entire experiment duration. 

In Trial Two, animals were moved to the experimental paddock on Study Day One, where they remained for the duration of the trial. Due to this gap between sensor attachment and entry to the paddock, valid data recording commenced from 1100 h on Study Day One. 

### 2.3. Observation

For Trial One, ewes were observed from 630 h to 1230 h and 1530 h to 1800 h (±30 min) for the entire experimental period (14 days). For Trial Two, observations were conducted from 0730 h to 1230 h and 1330 h to 1730 h (±30 min) for the entire experimental period (15 days). Observations were conducted for the purpose of recording lambing time, via the use of binoculars. Ewes were also fitted with identification “bibs” with unique colour/number combinations to allow the observer to differentiate individual ewes from a distance.

Time of lambing was recorded to the nearest hour where possible. Lambing was defined as the time in which the lamb was fully expelled. Hour records were rounded down, i.e., lambing events at 1301 h and 1359 h would both be recorded within 1300 h. If ewes lambed during the observational period, but the actual birth was not able to be observed (e.g., if ewes were hidden from view), the hour of birth was recorded within a maximum 2 h window. If this could not be determined, the record was discarded. Overnight lambing’s were not observed and were therefore excluded.

### 2.4. Data Management and Analysis

After each trial, the devices were removed, and data downloaded. GNSS tracking data were downloaded using the proprietary software (@Trip PC, Mobile Action Technology Inc., Taipei, Taiwan). Accelerometer data were downloaded using the proprietary software (OMGUI, Axivity Ltd., Newcastle, UK). All data were processed and analysed using the statistical software R [38]. Weather data from the on-farm weather station were also downloaded for the study period. The datasets for each trial were kept separate at all times.

### 2.5. GNSS Data

After download, the GNSS data were checked for fidelity. Any locations that had not been correctly logged (i.e., locations with a latitude and longitude of zero) were removed. Due to differences in the logging intervals between the trials (3 min Trial One; 2 min Trial Two), the GNSS data were interpolated to 5 min intervals. This interval was chosen as it was considered a more reasonable frequency for commercial application where battery life may be limited [39] and has been previously applied in sheep [4,40] and cattle [41,42]. This process was conducted by interpolating the existing GNSS tracks to a common time interval (5 min) using the redisltraj function in the R package adehabitatLT [43]. 

After interpolation, the distance and speed between successive locations were then calculated [4]. Speeds over 3 m/s were removed because these positions were likely inaccurate [6]. The distance, time and speed between successive GNSS locations were then recalculated and a moving window average of speed based on the two locations prior to and following the point of interest (i.e., five locations in total) were calculated.

To create a measure of the spatial relationship between each ewe and others in the flock, the distance between each ewe and each of her peers was determined. The straight-line distance between the GNSS locations for each ewe-pair was calculated using the “Vincenty (ellipsoid)” method [44]. Once the distance between each ewe-pair was calculated, values were averaged to calculate the mean distance to peers (MDP). The closest peer (CP; i.e., the smallest distance between ewes) was also recorded.

To calculate the spatial landscape utilisation of each ewe, the minimum convex polygon (MCP) was calculated for each ewe for every hour of the trial. MCP is a standard method for home range estimation [45]. To ensure MCP was not overestimated, the GNSS data were further processed to remove any locations outside of the paddock boundaries + 10 m (mean location error of i-gotU device < 10 m [46]).

### 2.6. Accelerometer Data 

After download, raw accelerometer data were processed according to the methods outlined in [10,33]. Briefly, a number of features were extracted from the raw X-, Y- and Z-axis values (see [10] for details). Features were calculated using two epoch lengths (10 s and 30 s). After feature extraction, previously developed ML algorithms [10] were used to classify the animal’s behaviours. Classification was conducted in three ways: (i) detection of specific behaviour (grazing, standing, lying and walking); (ii) detection of general activity (active or inactive); (iii) detection of posture (prostrate or upright).

### 2.7. Integrating GNSS, Accelerometer and Weather Data

Following raw data processing, the GNSS and accelerometer data sets were each summarised on an hourly basis and then integrated together with the weather data (Table 1). These summaries, and the selected features, are discussed in detail in the following sections (Section 2.7.1, Section 2.7.2 and Section 2.7.3). Hourly summaries were chosen to minimise data processing requirements while still allowing for detection at a relatively fine temporal scale. The use of hourly summaries also reflects previous work [4,6,33]. In the context of simulating a commercially relevant online model, hourly detection was also thought to represent a reasonable time frame in which a producer might be made aware and respond to any alerts developed.

#### 2.7.1. Features Derived from Prior Research

A number of key features for the GNSS and accelerometer data were selected due to their performance in previous research [4,6,33] or hypothesised as having potential in an integrated approach. For the GNSS data, key features were: (i) mean speed (MeanSp); (ii) minimum speed (MinSp); (iii) maximum speed (MaxSp); (iv) MDP; (v) CP and (vi) MCP. These features were based on previous work [4,6].

For the accelerometer data, key features were as follows: (i) the proportion of each hour spent performing mutually exclusive behaviours (grazing, standing, lying and walking); (ii) the proportion of each hour spent active; (iii) the number of times each individual changed their posture (i.e., upright to prostrate and vice versa) within an hour. These features were based on previous work [33]. 

#### 2.7.2. Peer-Based Features Comparing the Individual to the Flock

Given the gregarious nature of sheep [47], additional metrics were included in the integrated dataset to allow for concurrent assessment at an individual and flock-level. In an example outlined in [33], ewe walking behaviour was not only shown to increase at parturition, but also during periods of normal flock management (e.g., movement between paddocks). Based on this, it was decided that monitoring at both an individual and flock-level was necessary, noting that changes in behaviour of a single ewe would more likely indicate parturition, whereas broader changes to the flock would suggest a whole-flock change [33]. Thus, additional features were included comparing each ewe’s individual feature values at a given point in time to the mean value of all other animals at this time. These features were calculated as a percentage difference from the mean (i.e., percentage increase or decrease) and denoted “Name.Mean”, where “Name” refers to the feature of interest (see Table 1 for details).

#### 2.7.3. Temporal Comparison of Features 

To enable temporal comparison of features, the percentage increase or decrease in each feature was compared at key time intervals. Specifically, the percentage change between the current hour and the previous hour (Hour-1: denoted “Name.1 h”) or the current hour and 24 h previous (Hour-24: “Name.24 h”) was calculated. Inclusion of these time-based calculations was considered important to ensure temporal associations in behaviour were accounted for in the model. These calculations also allowed for a comparison of each individual against their own “baseline” to determine if significant changes in behaviour over time became evident (see Table 1 for details). Due to similarities between some derived features, a test for collinearity was conducted. Features with a correlation ±0.8 were removed from further analysis (Table 1).

### 2.8. Development of a Simulated Online Parturition Detection Model Using Machine Learning

ML algorithms are commonly used for pattern recognition and classification tasks [48] and have been successfully used in sheep behaviour research [9,10,49,50]. This process involves developing the algorithm with a training dataset and then testing it against an independent validation dataset. 

#### 2.8.1. Training Dataset

Data collected from Trial One were used as the training dataset and will henceforth be referred to as such. Once collated, the dependent variable on the training dataset was “labelled” to represent the behaviour state of the ewe (considered a binary state of either “lamb” or “non-lamb”). The process of labelling was as follows: the hour of birth (Hour 0) and one hour either side (Hour ± 1) were labelled as “lamb” (3 h in total). This was done to ensure that those animals that lambed earlier or later within the hour would still have an adequate representation of “lambing” behaviour included in the training dataset. Furthermore, the inclusion of multiple “lamb” hours per animal was important to increase the amount of available training data for this behaviour state for a more balanced dataset. Conversely, “non-lamb” hours were represented by the 24-h period for the third day prior to (Day 3) and third day after parturition (Day +3; 48 h in total). Only these days were selected to reduce the number of “non-lamb” hours in the training dataset. The use of data from three days prior to and following lambing was based on previous work [6,33], which suggests that most lambing-related behaviours do not commence until the day before (Day 1) or day of (Day 0) actual lambing. Balancing of behaviour representation in training datasets is important to ensure adequate machine learning can take place [51].

#### 2.8.2. Validation Dataset

Data collected from Trial Two were used as the validation dataset and will henceforth be referred to as such. The process of labelling the validation dataset was different and intentionally more specific compared to the training dataset. The hour of birth was labelled as Hour 0 and the hours surrounding Hour 0 were labelled numerically (±x hours) to represent the temporal association to the parturition event. For ewes where the hour of birth was known within a maximum 2 h window, the hour of birth was designated as the middle hour within the window, and the hours either side labelled as per the previous (i.e., a window of 1200 h–1400 h would designate 1300 h as Hour 0 (hour of birth), 1400 h as Hour + 1, etc.). If the middle hour fell on a part-hour, the hour was rounded down (i.e., 1330 h would round down to 1300 h).

#### 2.8.3. Part A: Simulated Online Parturition Detection ML Development and Evaluation

Support vector machine (SVM) classification was used to detect the binary ewe status (“lamb” or “non-lamb”). SVMs generate a hyperplane between observations to separate distinct classes [48], with the aim of maximising the distance between the observations and the hyperplane [52]. This ML algorithm has become popular in recent years due to its relative ease of application and high performance in real-world applications [52,53]. 

Leave-one-animal-out cross validation (LOOCV) was used to train and test the SVM. This process involved using all but one of the datasets to train the algorithm, with subsequent performance evaluation using the remaining dataset. During each training iteration, the data were pre-processed to “centre” and “scale”. The tuning cost (“C”) parameter was also adjusted using a grid-based search. This process was repeated for all animals to enable selection of the best C value.

Based on the first objective of this study, to determine which sensor systems and features provide the most useful information for lambing detection, feature selection was also conducted throughout this training process. To do this, a receiver operating characteristics (ROC) curve analysis was conducted using the varImp function from the caret package [54]. This function applies ROC curve analysis to each feature, calculates the resulting area under the curve and uses this area as a measure of feature importance between 0 and 100 [55]. Only features with an importance “score” over 75 were retained for algorithm training to reduce the complexity and computational requirements of the SVM as this is considered a limiting factor to commercial application. A similar approach has been reported in [24], where a single feature was incorporated into an online algorithm to minimise energy consumption. In that paper, the authors state that while including additional features can improve accuracy, their inclusion should be conducted under a cost-benefit approach given the computational costs of complex models [24].

Once trained, performance statistics for the SVM were calculated including: Kappa value, precision, recall (sensitivity) and the Matthews correlation coefficient (MCC). The Kappa value compares the observed accuracy with random accuracy and is considered informative in unbalanced samples such as in the current study [56]. Precision and recall are also useful for unbalanced samples where the focus is on detection of the smaller class [57]. MCC is widely used in bioinformatics for unbalanced classification [58], providing a score between −1 and 1, where 1 indicates prefect prediction, 0 indicates random prediction and −1 indicates total disagreement. Precision, recall and MCC were calculated using the following equations:

(1) $$\mathrm{precision}=\frac{TP}{\left(TP+FP\right)}$$

(2) $$\mathrm{recall}=\frac{TP}{\left(TP+FN\right)}$$

(3) $$\mathrm{MCC}=\frac{TP\times TN-FP\times FN}{\surd \left(TP+FP\right)\left(TP+FN\right)\left(TN+FP\right)\left(TN+FN\right)}$$

where *TP*, *TN*, *FP* and *FN* refer to true positive (correct classification of “lamb”), true negative (correct classification of “non-lamb”), false positive (incorrect classification of “non-lamb” as “lamb”) and false negative (incorrect classification of “lamb” as “non-lamb”), respectively. Accuracy was not calculated due to the imbalanced nature of the dataset [59]. 

#### 2.8.4. Part B: Validation of the Parturition Detection Model

Based on the second objective of this study, the final algorithm was applied to the independent validation dataset to detect the day and hour of lambing. To simulate an online situation, the first hour where lambing detection occurred was recorded and compared to the known time of birth. Detection success was assessed across two timeframes: firstly, if it was within ±1 h of the recorded hour of birth; secondly, if it was within ±3 h of recorded hour of birth. These two different levels were implemented to make it possible to identify both pre- and post-parturient behaviours, which are known to change in the hours just prior to or following lambing [33]. For example, in a study by Arnold and Morgan [60], pre-lambing maternal interest and behavioural changes associated with parturition were found to increase most significantly between 180 and 120 min prior to birth. A broader detection window was also important to allow for the complete length of labour (approximately 65 ± 9 min [61]), and detection of early post-parturient behaviour, such as the tendency to remain at the birth site for up to 5 h (mean of 2 h) [62]. If lambing detection occurred within ±3 h of known birth, evaluation ceased, and the model was no longer applied to that animal. If ewes did not have a correct detection within ±3 h, the evaluation continued until the known day of birth, after which, further lambing detection was also ceased. This enabled the evaluation of the likely number of false positives that were generated.

## 3. Results

### 3.1. Data and Lambing Records

A summary of the sensor and lambing records is presented in Table 2. In each year, a number of devices failed to record data and were excluded. In addition, one ewe prolapsed during the 2017 trial and was removed from the data set. Ewes that gave birth overnight or did not give birth during the experimental period were also excluded due to uncertainty of the exact time of birth.

### 3.2. Weather Records

During Trial One (training dataset), temperatures ranged from 3.8 °C to 22.3 °C and total rainfall was 85.6 mm. Average daily wind speed was 9.2 km/h with an average gust speed of 21.7 km/h. Average solar radiation was 104.9 w/m^2^/h.

During Trial Two (validation dataset), temperatures ranged from 0.7 °C to 21.6 °C. Average daily wind speed was 7.1 km/h with an average gust speed of 18.0 km/h. Average solar radiation was 176.0 w/m^2^/h. There was no rainfall during this period.

### 3.3. Part A: Simulated Online Parturition Detection ML Development and Evaluation

#### 3.3.1. Feature Importance

**Figure 1:**
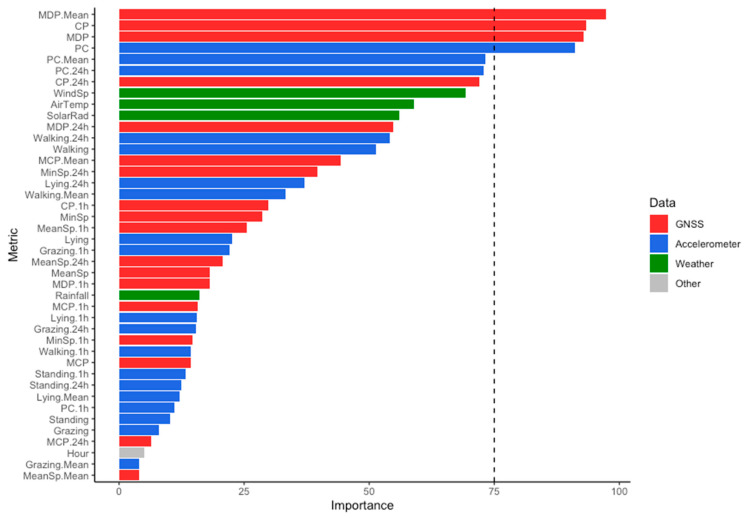
Feature importance for the integrated dataset determined by receiver operating characteristics (ROC) curve analysis. Data types are: GNSS-derived (red); accelerometer-derived (blue); weather (green) and other (grey). Only those metrics with an importance “score” above the chosen threshold (dashed line) were used in the ML. Refer to Table 1 for metric definitions.

Using the ROC curve analysis (Figure 1), the feature with the highest importance for differentiation between lambing and non-lambing animals was MDP.Mean (i.e., the MDP of the ewe compared to the average MDP of all others in the flock, expressed as a percentage). This was closely followed by CP and MDP (both expressed in metres). These features are all GNSS-derived. The most important accelerometer-derived features were PC (hourly number of posture changes), followed by PC.Mean (i.e., PC of the ewe compared to the average PC of all others in the flock, expressed as a percentage) and PC.24 h (i.e., PC of the ewe in the hour of interest compared to the same hour in the previous day, expressed as a percentage). Three weather features (wind speed, air temperature and solar radiation) were within the top 10 most important features. Hour of the day was not an important feature for the purposes of differentiation.

As depicted in Figure 1, it is clear that both GNSS and accelerometer sensors provide the most useful information for identification of lambing. Specifically, four features emerged as having an importance “score” over 75 and were retained in the final model: MDP.Mean, CP, MDP and PC. Although the original objective of the study was to examine an integrated sensor approach for parturition detection, due to the apparent importance of the GNSS metrics in the ROC curve analysis, a second SVM was developed at this stage to examine the benefits of using GNSS data alone.

#### 3.3.2. ML Evaluation

The two SVM models (the integrated SVM and GNSS SVM) were evaluated by LOOCV using the training dataset from Trial One. The integrated SVM performed slightly better than the GNSS SVM, with a higher Kappa (0.4), recall (48%) and MCC (0.6) compared to the single sensor dataset (Kappa: 0.3; recall: 33%; MCC: 0.5). The GNSS SVM demonstrated a higher precision (83%) compared to the integrated model (71%). Overall, the integrated SVM demonstrated a higher number of true positives (*n* = 10) compared to the single sensor (*n* = 7 true positives). Based on the performance of the integrated model, and due to the original objectives of understanding the value of integrated sensor systems, only the integrated model was selected for later validation using the Trial Two data.

**Figure 2:**
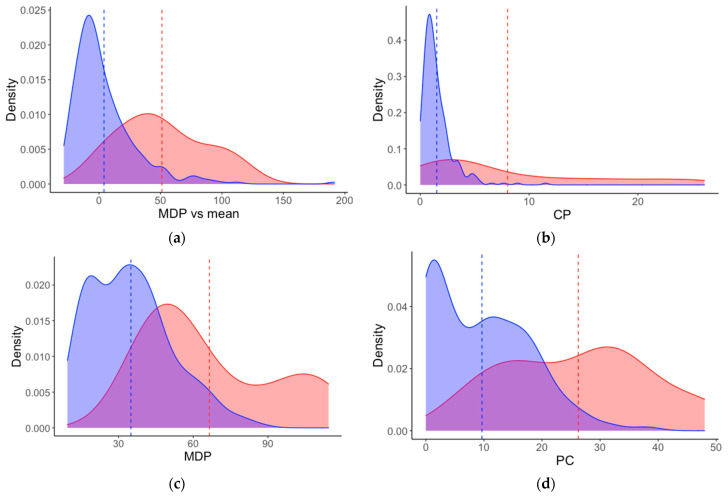
Density plots of the four most important features (determined by ROC curve analysis) for differentiation between lambing (red) and non-lambing (blue) animals using the training dataset (from Trial One). Features are: (**a**) MDP.Mean—Mean of mean distance to peers (for all peers); (**b**) CP—closest peer (for individual animal); (**c**) MDP—mean distance to peers (for individual animal); (**d**) PC—posture change (for individual animal). Mean lines are also shown (lambing: red dashed line; non-lambing: blue dashed line). The key characteristics of all features are the higher mean values and wider distributions when sheep are lambing.

Summary statistics (Table 3) and density plots (Figure 2) were generated for the training dataset to assist in understanding of the SVM classification process. As shown in Table 3, lambing animals displayed an increased level of social isolation compared to non-lambing animals, both in terms of actual distance (MDP) and when this distance was compared to the mean of the flock (MDP.Mean). This pattern was also evident for CP, with lambing animals being a mean distance of 8 m from their closest peer compared to non-lamb animals at 1.5 m. Frequency of changing posture also increased at lambing (mean 26.2 and 9.7 changes per hour for lambing and non-lambing animals, respectively).

Although all four features are dominant predictors for lambing, there is still some obvious overlap between the two behaviour states (Figure 2). For example, across all features, the maximum non-lamb values were consistently higher than the mean lambing values for each particular feature. This may contribute to inaccuracies in detection and highlights the potential to further refinement. This is explored further in Section 3.4.

### 3.4. Part B: Validation of the Parturition Detection Model

To explore if the integrated SVM could alert for parturition events as they occurred sequentially over time, the final model was tested against the independent validation dataset from Trial Two (Table 4). To simulate a near-real-time system where data would be made available in an incremental fashion, only the first hour of lambing detection was recorded and compared to the known time of birth. If this initial detection alerted too early (i.e., false positives before the actual birth event), the model was applied up until the actual day of birth to determine if later detection of the event would still occur.

Three animals (from a total of 11; 27%) had the first lambing alert within ±1 h of the known lambing hour. No additional animals had the first lambing alert within ±3 h. Seven ewes (64%) had a number of false positives (range: 1–28; mean: 8) prior to correct detection. One animal (Animal 6) did not have any alerts within ±3 h of known lambing hour. The closest alerts for this animal were Hour −6 and Hour +4.

While this model was able to alert to all but one parturition event, this high rate of detection comes at the cost of numerous false positive alerts (*n* = 64). To explore a second scenario under which false positives were less acceptable, a simple modification was applied. This basic change required identification of at least two consecutive “lamb” hours before an alert was generated. The results of this process are presented in Table 5.

Four animals (from a total of 11; 36%) had the first alert within ±1 h of the known lambing hour. An additional two animals had the first alert within ±3 h of the known lambing hour (6 in total within ±3 h; 55%). Three ewes had false positives (range 1–12; mean 5.5) with an accurate later detection within ±3 h of birth. Two ewes did not have any alerts occur within ±3 h of birth (Animals 6, 7). For these animals, the closest alerts were Hour −16 and Hour +5 (Animal 6) and Hour +9 (Animal 7).

#### Misclassification—Why Is It Occurring?

**Figure 3:**
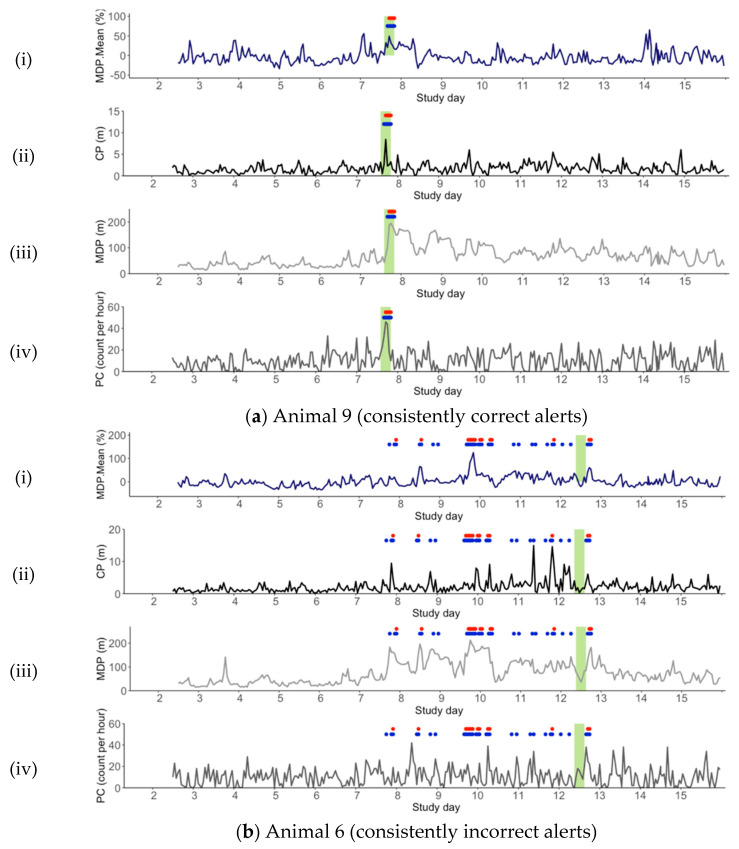

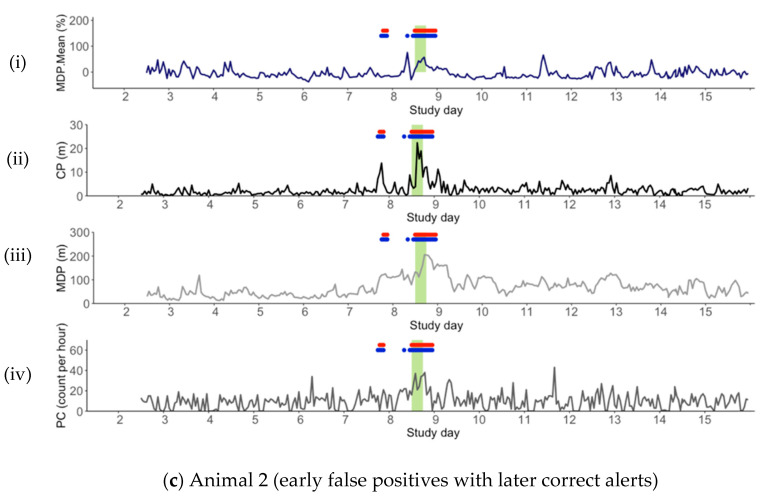
Individual datasets for Animal 9 (**a**); Animal 6 (**b**) and Animal 2 (**c**) (Trial Two). Features include (**i**) MDP.Mean (blue); (**ii**) CP (black); (**iii**) MDP (light grey); (**iv**) PC (dark–grey). Though the model was applied as a simulated online scenario, data is presented for all study days to enable visualisation of broader patterns. Alerts are shown for two scenarios: hour of alert (blue circles) and two consecutive ‘lamb’ hours required before alert (red circles). Alerts are included up to the day of birth for both scenarios. The hours of known lambing (Hours ± 3) are included as pale green shading.

To further explore reasons for misclassifications and to understand how the SVM used the data for lambing event detection, the individual datasets of three animals in the validation dataset were plotted (Figure 3). Lambing alerts and the period of the actual birth event were also plotted. The three chosen animals represent those that were consistently correct (Animal 9), consistently incorrect (Animal 6) or had early false positives but were later correct (Animal 2).

## 4. Discussion

This study represents the first reported attempt to integrate data from multiple sensors, both on-animal systems and weather data, for the purpose of parturition detection in pasture-based sheep. A number of studies have reported on the relationship between individual sensors and parturition [4,5,6,33,63,64]. However, none have attempted to explore how these data might be used in the context of developing a near-real-time lambing detection system that might be of value in commercial production systems.

### 4.1. Feature Importance for Lambing Detection

The four features most important for lambing detection were derived from GNSS (MDP.Mean, CP, MDP) and accelerometers (PC) and highlight the importance of these sensor types for lambing detection. Overall, ewes demonstrated an increased level of social isolation at lambing compared to non-lambing animals (Table 4 and Figure 2). This was evidenced by an increase in MDP.Mean, MDP and CP and supports published reports of ewe social isolation at parturition [4,6,65,66]. Increased frequency of changing posture was also exhibited by lambing ewes, with the mean number of hourly changes increasing almost 3-fold, from 9.7 to 26.2 (Table 4 and Figure 2). Again, this is consistent with published literature [61,67], and may indicate the onset of general restlessness associated with lambing.

Based on previously reported limitations of GNSS behaviour monitoring at an hourly scale [4,6], the reported importance of many of the GNSS-derived variables was initially unexpected. In [6], no significant differences in hourly MDP were found in the 12 h surrounding lambing. However, in the current study, this feature was ranked as the third most important for discrimination between lambing and non-lambing animals. In addition, in [6], GNSS was only shown capable of detecting the day but not the hour of lambing, whereas in the current study, GNSS-derived metrics were amongst the most important features identified (Figure 1). This disparity may reflect a difference in the methodologies of the two studies. In [6], the statistical comparison at an hourly scale was restricted to only 12 h around parturition. In contrast, in the current study the training dataset used values at lambing (±3 h) and compared them to non-lambing values collected 3 days either side of parturition. Indeed, in a second analysis in [6], broader changes in daily behaviour were found to indicate parturition, including MDP which increased from two days prior to birth. Thus, it appears that when compared to hours closer in time (as in [6]), there is a limited capacity to detect broader changes in behaviour that may occur over many days. Conversely, when behaviour is compared to hours more distant in time (as in the current study), the behaviour of ewes is detectably different.

In addition to the differences in methodology, the remaining GNSS features applied in the final model (MDP.Mean and CP) are novel features that, to the best of our knowledge, have not yet been reported for sheep using GNSS data. As previously noted, given the gregarious nature of sheep [47], measurements that assess flock-level behaviour change are important to differentiate individual changes in behaviour from the group. In contrast to previous literature that does not support the use of GNSS to detect hourly behaviour changes associated with parturition [4,6], the results of the current study suggests GNSS has notable monitoring ability, either in isolation or when integrated with an accelerometer. In the current study, the addition of Mean.MDP adjusts for changes in MDP that are the result of herd behaviour, which may be impacted by a number of factors, for example weather [18,66], forage quality [68,69] and social dynamics [70]. It is important to note that measurements of social activity using on-animal sensors are not limited to GNSS. Proximity loggers represent another sensor type that can provide this information, often in a smaller size with lower power requirements [25,71,72]. Referring to commercial platforms seeking to operationalise this research, it may be worthwhile exploring the substitution of a proximity sensor for a GNSS. However, this substitution may introduce limitations, given that it will result in the loss of some key functionality, particularly where the location data from the GNSS may be critical for the producer to actually respond to a lambing alert and find a ewe in an extensive landscape.

The reduced importance of accelerometer features in this analysis was also unexpected. This was particularly true for features related to walking behaviour, which have been previously reported as a powerful predictor of lambing [33]. The key accelerometer features identified were those related to posture change (PC, PC.Mean, PC.24 h), although only PC met the required threshold for inclusion. Given that accelerometers are generally small devices that can be easily applied to an animal [73], their integration into a commercial-grade device warrants further investigation. It should be noted that the method of detecting PC in this study required a significant level of data handling prior to the ML classification. More explicitly, the raw data had to be classified using previously developed ML models [10], after which hourly summaries of PC could be calculated and applied in the current model. This was considered to be essential as the actual process of ewes changing their posture was not adequately observed for ML training [10], and thus classification into two distinct postures was required before frequency of PC could be determined. Furthermore, classification into explicit behaviour allowed for comparison with known changes in parturition and assisted in the interpretability of the model. However, given the constraints of battery life and processing power in commercial situations [24], further exploration of posture change detection should be undertaken. For example, using metrics derived from the raw data such as movement variation (MV) or standard deviation of an accelerometer axis (SDX; SDY; SDZ; [9,10]). In previous work [10], MV, SDX and SDY were consistently identified amongst the most important predictors for classification of behaviour, general activity and posture. Thus, it is possible that use of these metrics may have similar predictive power when applied for parturition detection and should be considered in future studies.

The ROC curve analysis found weather features had only moderate importance for detection of parturition, particularly wind speed, air temperature and solar radiation. Sheep are known to have two major grazing episodes that are highly correlated to sunrise and sunset [74]. Weather is known to disrupt these patterns such as, for example, reduced grazing range in hot weather [18]. Weather is also known to impact social activity, particularly rising temperature and rainfall, which both result in increased social contact [70]. It is likely that the findings of the current study reflect the relatively mild weather conditions experienced, which may not have been extreme enough to have an impact on the ewe’s behaviour. Despite not playing an important role in the current study, inclusion of weather features in future models may still be warranted, particularly where more extreme weather events are experienced. 

### 4.2. Detection of Parturition and Implications for Commercial Application

The use of sensor technology in a commercial environment necessitates the development of near-real-time information transfer. As the challenges associated with implementation are still widespread, SOB devices have been applied in the current study as a proxy for simulated online application. In the current study, 27% (*n* = 3) and 55% (*n* = 6) of animals had an accurate lambing detection within ±3 h of birth with no prior false positives, depending on the detection criteria used (i.e., first hour of alert (Table 4) compared to two consecutive hours of alert (Table 5)). In a real-life scenario, it is unlikely that the model would automatically terminate as soon as the first alert occurs, instead requiring direct confirmation (or rejection) from the producer that lambing has (or has not) occurred. For this reason, inclusion of animals with initial false positives and later accurate alerts is also reasonable. Based on the latter, the results of the current study are encouraging, with 91% (*n* = 10; Table 4) and 82% (*n* = 9; Table 5) of lambing events successfully detected, depending on the detection criteria used. The models applied in the current study are not true real-time detection algorithms, as they require the collection of an entire hour’s worth of data before summary and detection can occur. However, current methods of lambing detection are usually based on visual observation, which may increase the risk of mismothering [28]. Thus, despite not being a true real-time model, successful remote detection of lambing within ±3 h could significantly increase the efficacy of ewe surveillance and may be useful for improving both production and welfare outcomes.

When considering the end-use of these models, it is important to understand how they may be applied in a real-life setting. For example, though the results of Table 4 indicate the ability to detect approximately 91% of birth events within a 3 h window using the first hour of alert, the high rate of detection was also accompanied by a high rate of false positives (*n* = 64). If this were applied directly in a commercial situation, it would correspond to a large number of false alarms for every correct alert. In situations where the animals represent a higher economic value (e.g., seed stock breeding animals), an increase in false positives may be tolerable if all events of interest are identified. In contrast, in most commercial production systems where the value of individual animals is lower, producers may prefer to reduce the number of false positives at the expense of potentially missing some events of interest [75].

As shown in Table 5, inclusion of the simple requirement for two consecutive lambing alerts decreased the number of false positives from 64 to 22. This scenario also narrowed the window of detection, with a further one and two animals having the first alert within ±1 h (Animal 5) or ±3 h (Animals 1 and 11), respectively. However, the restriction did increase the overall failure to detect a lambing event from one (Table 4) to two (Table 5). Practical application of the latter model might be found in a commercial production system where individual animal monitoring is less valuable and where refining flock-scale management brings economic return. For example, a producer may choose to be alerted when the flock has commenced lambing, applying this knowledge to initiate a flock-wide physical monitoring program (i.e., visual observation). This may be useful for flocks without adequate breeding records or if the flock are at known risk of adverse parturition events such as dystocia and/or prolapse [30]. Another example application might be the use of flock-level alerts for warning of increased lambing numbers, especially if the lambing events are occurring during periods of increased predation or adverse weather. In the current study, we have modified the model to sit at the end of two extremes and there is likely a mid-point where the applications are optimised. Exactly how the model sensitivity should be refined requires ample thought and should be contemplated in further research. This has also been discussed by Dominiak and Kristensen [75] where customisation of detection models is advised depending on two things: firstly, the priorities of the producer; secondly, the purpose of the model application (e.g., cost optimisation vs. health or welfare improvement). 

### 4.3. Understanding the Limitations and Reasons for Model Failure 

A key consideration for successful commercial application, is the ability of a detection model to generalise across a number of individuals. However, based on the results of the current study and our understanding of the variability between individual animal behaviour [47,76,77], this remains a challenge. One of the major limitations of many ML algorithms is the inability to interpret their internal “rules” used to categorise data [48]. In the case of the current SVM, although the model has a relatively high accuracy for differentiation between lambing and non-lambing animals, the requirements for classification, including thresholds and/or the required number of features for alert cannot be easily determined. To explore the ML models further, three of the animals’ feature traces are reported in detail (Figure 3). Through this, we can make inferences as to how the ML might be working and identify potential reasons for model failure.

As an example of an individual animal for which the classification algorithm worked well, Animal 9 (Figure 3a) shows obvious peaks in the data at the time of lambing, particularly for CP and MDP. This suggests Animal 9 was distant from the main flock at the time of parturition (peak CP 8.4 m at Hour 0; peak MDP 194.9 m at Hour + 1). In contrast, the classification algorithm did not work well for Animal 6 (Figure 3b), and CP and MDP actually fell at the hour of lambing, suggesting the ewe was not separate from the flock at this time (peak CP 4.0 m at Hour −3; peak MDP 85.2 m at Hour + 3). Given that isolation behaviour was evident in the training dataset (Table 3), the ML appears to rely on this expected behaviour for correct alerts (Animal 9) and hence cannot identify lambing when this expected behaviour does not occur (Animal 6). This is further supported by the earlier peaks in CP and MDP for Animal 6 that correspond to early false positives. Of note, given that the MDP.Mean did not peak for Animal 9 at lambing, it appears that the ML does not require all three social metrics to increase for an alert to occur. 

Examination of the PC feature reveals a similar scenario. That is, for Animal 9 (Figure 3a), an increase in PC behaviour at lambing was evident, and was accompanied by correct lambing detection. In contrast, Animal 6 (Figure 3b) demonstrated decreased PC at the time of lambing, which again contributed to the model missing the event detection. For both Animal 9 and Animal 6, earlier peaks in PC behaviour were evident prior to parturition. However, for Animal 9, these were not accompanied by peaks in the remaining features, whereas for Animal 6, the increased PC behaviour was also accompanied by peaks in social isolation, ultimately resulting in a number of false positive alerts. Thus, while it appears that the model may not require all three measurements of social isolation for an alert to occur, the algorithm appears to be sensitive to changes in behaviour when they occur at the same time as other key fluctuations. Although the introduction of the stricter detection criteria did reduce these false positives somewhat, it does not mean that the more “unexpected” patterns of behaviour for individual ewes can be mitigated.

Animal 2 is an example of a ewe that displays early false positives followed by correct lambing alert. As shown in Figure 3c, false positives were evident on the day prior to birth (Study Day 7) due to a peak in both CP and MDP. This may reflect variable social activity of this ewe or it may demonstrate early social isolation and/or a time of birth site selection or nesting behaviour [29,61]. If this could be isolated, this behaviour could be used as powerful predictor of impending parturition, providing producers with the opportunity to act on an alert prior to the event occurring. However, as isolation at parturition is inconsistent in domestic sheep [29], the ability to generalise this behaviour across numerous individuals is unlikely.

### 4.4. Recommendations for Future Research

In this study, the method of data labelling was fixed for the training dataset between animals (i.e., Hours ± 1 labelled as “lambing”; 3 h in total). However, as parturient behaviour is known to vary between animals [76,77], this labelling protocol may have been too rigid for the natural inconsistencies that exist. Although the impact of labelling protocol is valid, it should also be noted that the variation in lambing behaviour may also be a product of normal diurnal changes (e.g., normal grazing patterns). For example, parturition records in this study were collected at various times throughout the observation period, depending on when the animals lambed. This means that ewes that gave birth during the normal peak morning or evening grazing periods were labelled identically (and thus indistinguishable) to those that lambed during normal periods of resting or rumination. Given that spatial behaviour is known to change throughout the day [74], it is possible lambing behaviour in this study is confounded by time itself, thus resulting in higher variability (and wider density plots) for the lambing day data (Figure 2). In contrast, the non-lamb data are represented by two 24-h periods, one 3 days prior to lambing and one 3 days after lambing, and thus will naturally contain data that are representative of the entire diurnal pattern. Further research should be conducted to determine the impact of the labelling protocol, potentially using detailed individual observations as a method of identifying the commencement of lambing behaviour. Research should also be conducted using data from ewes that lamb overnight and on a larger number of animals, to ensure the patterns in behaviour are consistent across a number of contexts. Finally, further research should be conducted to determine if the presence of “false positive” alerts prior to lambing are actually incorrect detections, or simply the identification of earlier onset of lambing behaviour or the presentation of different patterns of behaviour than previously reported. The use of this knowledge would also be beneficial to examine in problem-birth events and warrants further evaluation.

Although this study has presented an adequate method of simulating online parturition detection, application of this knowledge in commercial systems still requires further thought. For example, the use of embedded processing and edge computing has been suggested for commercial application, given the energetic costs of data transmission [24]. However, in the current study, the most valuable features for parturition detection were those that compared the individual ewe’s behaviour within the wider flock context (i.e., MDP, MDP.Mean, CP, PC.Mean) or relative to its own past behaviour (PC.24 h). In the latter instance, edge computing is valid as previous data could be stored on the device and used as a comparative metric to current readings. In the former example, however, comparison with other ewes is necessary and would require transfer of data to a central repository for parallel processing with all other devices. Given this, future research should investigate how the data can be condensed prior to transmission and still be useful for comparison to other ewes.

## 5. Conclusions

The outcomes support the use of integrated sensor data for ML-based detection of parturition events and lambing activity in grazing sheep using SOB data as a proxy for near-real-time detection. This is the first known application of ML classification for the detection of lambing in pasture-based sheep. Four main features generated from GNSS and accelerometer data were identified as the most useful for lambing detection: MDP.Mean, CP, MDP and PC. Using these features, information on ewe social activity and frequency of changing posture is used to detect if a lambing event has occurred within the previous hour. Though weather data were not used in the final model, all sensor types were well represented across the ROC curve analysis, thus highlighting the benefits of sensor integration. A surprising outcome of the current study was the success of applying the GNSS data for parturition detection in isolation, without the added integration of the accelerometer data. This suggests that application of GNSS over a longer time period and with novel comparisons to flock-level behaviour are important to adequately represent the value of this sensor type. 

In the current study, the ML models were able to detect lambing events with reasonable accuracy. This success depended on variation in individual animal behaviour and highlights the sensitivity of the ML model for detecting a change in key behaviours. Further research should consider the use of this model (or similar) for detection of adverse lambing events. This would have significant impacts on the ability to remotely monitor animal welfare using on-animal sensors and is a logical extension of the information presented in this paper.

## Figures and Tables

**Table 1:** Features provided from each sensor type, including the unit of measurement. Derived features are reported as absolute values per hour. Peer-based features are calculated as the percentage difference between the individual ewe and the mean of all other ewes in the flock. Temporal features are calculated as the percentage difference between the current hour and the previous hour (1 h) or 24 h previous (24 h). Features removed due to collinearity are in italics. FNP = Feature not progressed. NA = Not applicable.

Sensor Type	Derived Features	Unit	Peer-Based Features	Unit	Temporal Features	Unit
	Mean speed (MeanSp)	m/s	MeanSp.Mean	%	MeanSp.1 h/24 h	%
	Minimum speed (MinSp)	m/s	*MinSp.Mean* ^1^	*-*	MinSp.1 h/24 h	%
GNSS	*Maximum speed (MaxSp)* ^2^	*-*	*FNP* ^2^	*-*	*FNP* ^2^	*-*
	Mean distance to peers (MDP)	m	MDP.Mean	%	MDP.1 h/24 h	%
	Closest peer (CP)	m	*CP.Mean* ^3^	*-*	CP.1 h/24 h	%
	Minimum convex polygon (MCP)	%	MCP.Mean	%	MCP.1 h/24 h	%
	Time spent grazing (Grazing)	%	Grazing.Mean	%	Grazing.1 h/24 h	%
	Time spent lying (Lying)	%	Lying.Mean	%	Lying.1 h/24 h	%
Accelerometer	Time spent standing (Standing)	%	*Standing.Mean* ^4^	*-*	Standing.1 h/24 h	%
	Time spent walking (Walking)	%	Walking.Mean	%	Walking.1 h/24 h	%
	*Time spent active* ^5^	*-*	*FNP* ^5^	*-*	*FNP* ^5^	*-*
	Posture changes (PC)	Count	PC.Mean	%	PC.1 h/24 h	%
	Average air temperature (AirTemp)	°C/h	NA	NA	NA	NA
Weather data	Hourly rainfall (Rainfall)	mm/h	NA	NA	NA	NA
	Average wind speed (WindSp)	kph	NA	NA	NA	NA
	Average solar radiation (SolarRad)	w/m^2^/h	NA	NA	NA	NA

^1^ Removed from analysis due to collinearity with MinSp; ^2^ removed from analysis due to collinearity with MeanSp (no additional features calculated); ^3^ removed from analysis due to collinearity with CP; ^4^ removed from analysis due to collinearity with S; ^5^ removed from analysis due to collinearity with time spent grazing (no additional features calculated).

**Table 2:** Data and lambing records for the training (Trial One) and validation (Trial Two) datasets.

	Training	Validation
Animals at trial initiation	40	39
Animals with one or more failed devices	5	6
Complete datasets at trial conclusion	35	33
Excluded datasets	27 ^1^	22 ^2^
Day and hour of birth identified	8	9
Hour of birth known within a maximum 2 h window	0	2
TOTAL	8	11

^1^ Exclusion based on prolapse (*n* = 1) or unknown lambing time (overnight or outside of the experimental period; *n* = 26); ^2^ exclusion based on previous use in machine learning (ML) algorithm development (*n* = 12; [10]) or unknown lambing time (overnight or outside of the experimental period; *n* = 10).

**Table 3:** Summary statistics for the top four features of the training (Trial One) dataset (determined by ROC curve analysis).

Features	Lamb			Non-Lamb		
	Mean	Min	Max	Mean	Min	Max
MDP.Mean (%)	51.3	−3.8	118.9	4.1	−28.6	191.5
CP (m)	8.0	0.6	26.1	1.5	0	11.5
MDP (m)	66.5	37.6	114.4	35.0	9.5	87.5
PC (count)	26.2	6	48	9.7	0	38

**Table 4:** Application of the integrated support vector machine (SVM) to the validation dataset (Trial Two). Hour of first alert is expressed relative to the recorded hour of birth. Notations “X” and “X+” indicate the animal meets the criteria.

Animal	Hour of First Alert	False Positives (Prior to Actual Lambing)	First Alert within ±1 h (X) or ±3 h (X+)	Early False Positives with Later Accurate Alert ±3 h	Failed (No Alerts ±3 h)
1	−67	1		X	
2	−21	6		X	
3	0	0	X		
4	−1	0	X		
5	−43	1		X	
6	−114 (4.8 days)	28			X
7	−118 (4.9 days)	3		X	
8	−169 (7.0 days)	18		X	
9	−1	0	X		
10	−56	6		X	
11	−68	1		X	
TOTAL		64	3 (+0)	7	1

**Table 5:** Application of the integrated SVM to the validation dataset (Trial Two) with the additional criteria of requiring identification of at least two consecutive “lamb” hours before an alert was generated. Hour of first alert is expressed relative to the recorded hour of birth. Notations “X” and “X+” indicate the animal meets the criteria.

Animal	Hour of First Alert	False Positives (Prior to Actual Lambing)	First Alert within ±1 h (X) or ±3 h (X+)	Early False Positives with Later Accurate Alert ±3 h	Failed (No Alerts ±3 h)
1	+2	0	X+		
2	−20	3		X	
3	+1	0	X		
4	0	0	X		
5	0	0	X		
6	−110 (4.6 days)	12			X
7	+9	0			X
8	−141 (5.9 days)	6		X	
9	0	0	X		
10	−16	1		X	
11	+3	0	X+		
TOTAL		22	4 (+2)	3	2

## Data Availability

The data presented in this study are available on request from the corresponding author.
